# Tegaserod maleate exerts anti-tumor effects on prostate cancer via repressing sonic hedgehog signaling pathway

**DOI:** 10.1186/s10020-025-01080-1

**Published:** 2025-01-29

**Authors:** Maoping Cai, Shengdong Ge, Yaying Hong, Yao Chen, Yang-Zi Ren, Dacai Zhong, Mingkun Chen, Yuan Liu, Zhe-Sheng Chen, Ninghan Feng, Zhanghui Chen, Shan-Chao Zhao

**Affiliations:** 1 Department of Urology, The Fifth Affiliated Hospital of Southern Medical University, Guangzhou, 510920 Guangdong People’s Republic of China; 2 The Third Clinical College, Southern Medical University, Guangzhou, 510630 Guangdong People’s Republic of China; 3 Zhanjiang Institute of Clinical Medicine, Central People’s Hospital of Zhanjiang, Zhanjiang, 524045 Guangdong People’s Republic of China; 4 Department of Urology, Nanfang Hospital, Southern Medical University, Guangzhou, 510515 Guangdong People’s Republic of China; 5 College of Pulmonary and Critical Care Medicine, The 8th Medical Center of Chinese People’s Liberation Army (PLA) General Hospital, Beijing, China; 6 Department of Oncology, The First Affiliated Hospital of Guangzhou University of Chinese Medicine, Guangzhou, 510405 Guangdong People’s Republic of China; 7 Department of Urology, The Third Affiliated Hospital of Southern Medical University, Guangzhou, 510630 Guangdong People’s Republic of China; 8 Department of Pharmaceutical Sciences, College of Pharmacy and Health Sciences, St. John’s University, Queens, NY 11439 USA; 9 Department of Urology, Jiangnan University Medical Center, Wuxi, 214002 People’s Republic of China; 10 Wuxi School of Medicine, Jiangnan University, Wuxi, 214002 People’s Republic of China; 11 Department of Urology, Affiliated Wuxi No. 2 Hospital, Nanjing Medical University, Wuxi, 214002 People’s Republic of China

**Keywords:** Prostate cancer (PCa), Tegaserod maleate (TM), Sonic hedgehog (SHH) signaling, GLI2, Treatment

## Abstract

Prostate cancer (PCa) is a highly common type of malignancy and affects millions of men in the world since it is easy to recur or emerge therapy resistance. Therefore, it is urgent to find novel treatments for PCa patients. In the current study, we found that tegaserod maleate (TM), an FDA-approved agent, inhibited proliferation, colony formation, migration as well as invasion, caused the arrest of the cell cycle, and promoted apoptosis of PCa cells in vitro. In addition, TM suppressed the tumor growth in the cell-derived xenograft (CDX) mouse model in vivo. Mechanistically, TM exerted anti-tumor effects via downregulating GLI2, and its downtream targets, thus inhibiting the sonic hedgehog (SHH) signaling pathway. In brief, our findings demonstrated that TM effectively inhibited the activities of PCa cells by suppressing the SHH signaling pathway and provided a potential new agent for the treatment of PCa.

**Supplementary Information:**

The online version contains supplementary material available at 10.1186/s10020-025-01080-1.

## Introduction

Prostate cancer (PCa) is the most prevalent type of male malignancy and the main cause of cancer-related death among males globally (Siegel et al. 2022; Xia et al. 2022; Liu et al. 2022). Regardless of initial susceptibility to androgen deprivation therapy (ADT), the majority of patients eventually develop castration-resistant prostate cancer (CRPC) within 18–36 months of ADT (Cornford, et al. 2021; Jamroze et al. 2021). With advancements in medical technologies, various treatments such as chemical, endocrine, immune, and radical therapies for PCa have been developed (Yamada and Beltran 2021; Lu and Gao 2022). However, these treatments often lead to therapy resistance, resulting in low cure rates and poor prognosis (Cai et al. 2023; Ruiz de Porras et al. 2021). Therefore, there is an urgent need to explore novel strategies, and anti-cancer mechanisms to enhance treatment efficacy and provide new options for managing PCa, particularly CRPC.

Tegaserod maleate (TM) is a drug that selectively acts as a partial agonist on the 5-HT4 receptor and an antagonist on the 5-HT2B receptor. It has been clinically used for several decades in the treatment of patients with irritable bowel syndrome (IBS) due to its effects on the gastrointestinal (GI) tract (Scott and Perry 1999). In recent years, researchers identified a novel potential role of TM as an anti-cancer drug. Multiple studies have demonstrated that TM strongly inhibits the development of numerous malignancies, including acute myeloid leukemia, melanoma, breast cancer, and gastric cancer (Hong et al. 2023; Xie et al. 2022a; Liu et al. 2020; Wang, et al. 2022; Li et al. 2022a). Recently, studies have found that YTHDF1, an m6A reader protein, is overexpressed in PCa and plays an essential role in the progression as well as therapy resistance in PCa (Li et al. 2022b; Wang et al. 2023). Hong et al. identified TM as a potent YTHDF1 inhibitor (Hong et al. 2023). Consequently, we speculated that TM exerted profound inhibitory effects on PCa cells and verified it.

In this study, we identified TM as a potent drug to suppress PCa cells. TM efficiently repressed PCa cells proliferation, migration, and invasion while also promoting apoptosis. Transcriptomic and functional analyses revealed that TM led to the decrease of GLI2 and its target genes, thus inhibiting the sonic hedgehog (SHH) signaling pathway. Hedgehog (HH) proteins, which include SHH, desert hedgehog (DHH), and indian hedgehog (IHH), from *Drosophila* to humans, play critical roles in the evolution of organs and cells (Chen and Struhl 1996). HH binding to the patched receptor (PTC) in vertebrates reduces the inhibition of the co-receptor smoothened (SMO), activating downstream GLI transcription factors such as GLI1, GLI2, and GLI3. GLI1 is a transcriptional activator, while GLI2 and GLI3 comprise both activator and repressor forms (Jiang 2022). GLI3 undergoes proteolytic cleavage to provide GLI3R, a C-terminally truncated repressor form in the absence of HH, while GLI2 is targeted for inhibition. When HH is present, GLI3 cleavage into GLI3R is prevented by the ensuing signaling, which also stabilizes GLI2, allowing it to subsequently be N-terminally truncated into its activator form (GLI2A) (Briscoe and Thérond 2013). Generally, HH has a conserved effect of changing GLIs from repressors to activators, enabling coordinated transcriptional activities (Jiang 2022). In addition, SHH plays an essential role in the development of cancers including PCa (Datta and Datta 2006). Above all, we speculated that TM might serve as a potential new antagonist of the SHH signaling pathway, and be used to treat diseases caused by the activation of the SHH signaling.

In our study, we identified TM as a potent drug to suppress PCa cells. It efficiently inhibited PCa cells proliferation, migration, and invasion, and induced apoptosis in vitro. It also inhibited tumor development in vivo. Transcriptomic and functional analyses revealed that TM treatment led to the decrease of GLI2 and inhibition of the SHH signaling pathway, accounting for the underlying mechanism of its anti-tumor activity.

## Material and methods

### Cell culture and treatment

The ATCC (Manassas, VA, USA) provided the DU145, PC-3, LNCaP, 22RV-1, and RWPE-1 cell lines, which were then cultivated in accordance with earlier instructions (Yu et al. 2022; Xie et al. 2022b; Xiao et al. 2021). Prior to being used, cell lines were verified to be mycoplasma-free by short tandem repeat (STR) profiling. We did not employ cells that were beyond passage 20. Cells in experimental cultures were exposed to the TM doses listed (MCE, HY-14153A). Dimethyl sulfoxide (Sigma, S-002-M) was used as the vehicle for TM.

### Cell proliferation assay

3000 cells per well were seeded into 96-well plates with 100 μL of medium containing various experimental drug concentrations at 37 °C for the appropriate number of hours. The medium was then replaced with 100 μL of a solution containing 10 μL from the Cell Counting Kit (CCK-8; MCE, HY-K0301), and the plates were incubated for an additional two hours at 37 °C and 5% CO2. Ultimately, a Thermo microplate reader was used to measure the OD value at 450 nm.

In order to conduct the colony formation experiments, 1500 cells per well were seeded onto 6-well plates, and the cells were grown at 37 °C for 12 days using varying concentrations of TM as the vehicle. Finally, the cells were fixed with 4% paraformaldehyde (PFA) dyed with crystal violet, and photographed.

### Flow cytometry analysis

Using the Cell Cycle Staining Kit (KeyGEN, KGA512) and the associated procedure, the cell cycle analysis was carried out. In short, after 48 h of growth in 6‐well plates (10^6^ cells/well) with either vehicle or TM, the cells were collected and preserved for the night at 4 °C in 70% ethanol. Subsequently, the cells were collected for propidium iodide (PI) staining after the ethanol was removed. The cells were ultimately examined using a BD Biosciences C6 flow cytometer.

The apoptosis analysis was carried out in accordance with the relevant protocol using the Annexin V-PE/7-AAD Staining Kit (KeyGEN, KGA1018). In 6-well plates (10^6^ cells/well), cells were grown with vehicle or TM for 48 h at 37 °C. Following that, the cells were collected for 7-Aminoactinomycin D (7-AAD) staining and ultimately examined using a BD Biosciences C6 flow cytometer.

### Scratch assays and transwell experiments

Ibidi Culture-Insert (Ibidi GmbH, Münich, Germany) was used to set up the scratch assay. Cells were seeded into the culture-insert in 12-well plates. To reduce cell growth after the attachment of the cells, the culture-insert was taken out and the culture medium was substituted with media containing vehicle or TM containing 1% fetal bovine serum (FBS). Lastly, a light microscope was used to inspect and take pictures of the scratch after 0, 12, and 24 h. ImageJ was used to quantify cell movement.

Regarding the transwell migration experiment, 100 μL of media devoid of FBS was used to seed 4 × 10^4^ cells into the top chamber of 24‐well transwell plates (6.5 mm insert, 8.0 μm pores). To the bottom compartment, 600 μL of 10% FBS-supplemented media was introduced. A cotton swab was used to gently remove the cells from the chamber's top surface after 24 h. After that, the migrating cells on the chamber bottom were fixed with 4% PFA, stained with crystal violet and photographed.

As to the transwell invasion assays, 24 well transwell plates were filled with an upper chamber containing 100 μl of diluted Matrigel (BD), which was then incubated for two hours at 37 °C with 5% CO2. 100 μL of serum-free media was used to seed about 4 × 10^4^ cells into the top chamber. To the bottom compartment, 600 μL of 10% FBS-supplemented media was introduced. Using a cotton-tipped swab, the Matrigel and non-invading cells were carefully removed from the chamber's top surface after 24 h. After that, the cells on the chamber bottom were fixed and dyed. For quantification, three randomly chosen fields from each chamber were photographed under a light microscope.

### Plasmid construction and transfection

Plasmid construction and transfection were performed as stated previously (Yan et al. 2023). The PCDH vector's NheI and EcoRI restriction sites were subcloned with the full-length coding sequences (CDS) of GLI2. DNA sequencing served as validation for each plasmid. As directed by the manufacturer, Lipofectamine 3000 (Invitrogen Life Technologies®, Carlsbad, CA, USA) was used to transfect plasmids. GLI2 expression was assessed by Western blotting using anti-GLI2 (Abclonal, A16863) antibodies and RT-qPCR.

### RNA isolation, RNA-sequencing (RNA-seq), RT-qPCR and Western blotting

The methods previously described (Hong et al. 2023) were followed for RNA isolation, RNA-seq, real-time quantitative polymerase chain reaction (RT-qPCR), and Western blotting. TRIzol Reagent (Invitrogen) was used to extract total RNA from PCa cells treated with vehicle or TM for 48 h, and RNA seq analysis was performed by Hangzhou Kaitai Biotechnology Co., Ltd. (Hangzhou, China).

Using the Evo M-MLV kit (AG11606), 1 μg of RNA was transcriptionally converted to complementary DNA (cDNA) for RT-qPCR. SYBR Green reaction mix (AG11732) and a Light Cycler 96 System (Roche, Basel, Switzerland) were used for the qPCR. Ct, the cycle threshold, was used to compute the relative expression using the 2^−ΔΔCt^ method. Supplementary Table 1 contains a list of sequences of all the particular primers.

About Western blotting, the protease inhibitor cocktail-containing RIPA Lysis and Extraction Buffer (Fudebio, FD008) was used to lyse the cells. After that, proteins were separated by SDS–polyacrylamide gel electrophoresis and put onto membranes made of nitrocellulose. The membranes were blocked for 1 h at 25℃ with 5% fat-free milk, and then they were treated with primary antibodies for an overnight period at 4 °C: GLI2 antibody (Abclonal, A16863), PTCH1 antibody (Abclonal, A0826), SUFU antibody (Abclonal, A13429), SMO antibody (Abclonal, A3274), SHH antibody (Abclonal, A18863), Cyclin D1 antibody (Abclonal, A11022), Cyclin D2 antibody (Abclonal, A1773), Cyclin E1 antibody (Abclonal, A22461), CDK4 antibody (Abclonal, A2352), GLI1 antibody (Abclonal, A14675), c-Myc antibody (Abclonal, A1309), Bcl-2 antibody (Abclonal, A0208), GAPDH antibody (CST, #2118), β-actin antibody (CST, #4970) and β-tubulin antibody (CST, #2146). The membranes were then incubated with HRP-conjugated, anti-rabbit secondary antibody (Sigma, GENA934) for 1 h at room temperature.

### Mouse model and in vivo treatments

The animal care committee of the Central People's Hospital in Zhanjiang authorized the animal research (approval number: ZJDY2023-61). Subcutaneous injections of 1 × 10^6^ DU145 or PC-3 cells were given to male athymic nude mice (Jiangsu JICUI Laboratory Animal) that were five weeks old. Mice were injected intraperitoneally with vehicle or TM (2.5 mg/kg) every two days for seven days after implantation. PEG 300 and Tween-80 were used as the TM solvents for the in vivo investigation. Following medication delivery, the tumor volume and body weight of nude mice were monitored every two days for the following three weeks. The formula V = L × W2/2 (V = volume, mm3; L = major axis length, mm; W = minor axis length, mm) was used to estimate the tumor volume. Using a digital caliper, the length of the main and minor axes was measured. Following a three-week period of medication treatment, the tumors were removed, quantified, photographed, embedded in 4% PFA, and sectioned into paraffin.

### Immunohistochemistry (IHC) assays

We used the paraffin slices of the xenograft tumor for IHC. The following tools were used for IHC: HRP-conjugated goat anti-rabbit IgG (Fudebio, FDR007), GLI2 antibody (Abclonal, A16863), cleaved caspase-3 antibody (CST, 9661), Ki67 antibody (CST, #9027), SHH antibody (Abclonal, A18863) and Instant Immunohistochemistry Kit I (KeyGEN, KGOS300). Under a light microscope, stained slices were viewed and photographed.

### Statistical analysis

Software for graphics was GraphPad Prism 9, and statistical analysis was done using SPSS. The mean ± SD was used to represent the experimental outcomes. For statistical analysis, the student's t test was used. P-values less than 0.05 (*), less than 0.01 (**), or less than 0.001 (***) were regarded as statistically significant.

## Results

### TM inhibited the proliferation and promoted apoptosis of PCa cells in vitro

**Fig. 1:**
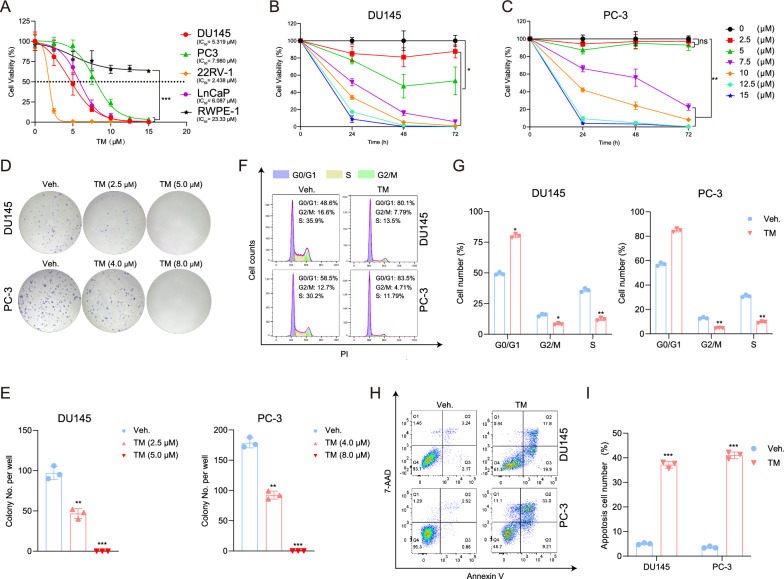
Tegaserod maleate (TM) inhibited proliferation of PCa cells. **A** Cells were treated with TM (0, 2.5, 5, 7.5, 10, 12.5, 15 μM) for 48 h, and subsequently, cell viability was detected through CCK-8 assays. Statistical analysis was performed between each PCa cell line and RWPE‐1. **B**, **C** DU145 and PC-3 cells were treated with TM (0, 2.5, 5, 7.5, 10, 12.5 and 15 μM) for 0, 24, 48 or 72 h, and then, cell viability was determined by CCK‐8 assays. Statistical analysis was performed between cells treated with 0 μM TM and each of the other drug concentration groups. **D**, **E** Colony formation assay on DU145 treated with TM (0, 2.5 and 5 μM) and PC-3 cells treated with TM (0, 4 and 8 μM) respectively. **F**, **G** Cell cycle distribution of DU145 and PC-3 cells incubated with 5 μM and 8 μM TM, respectively, or vehicle for 48 h. **H**, **I** Flow cytometry assays of the apoptotic percentage (including viable and non‐viable apoptotic cells) in TM‐ and vehicle‐treated DU145 (5 μM TM) and PC-3 (8 μM TM) cell. Unpaired t test was used for the statistical analysis. *P < 0.05; **P < 0.01; ***P < 0.001; ns, no significance. Data are presented as mean ± SD of at least three independent experiments

To investigate whether TM inhibited PCa cells growth, we first performed CCK-8 assays on PCa cells including DU145, PC-3, 22RV-1, LNCaP, and normal epithelial cell (RWPE-1), treated with TM to detect their viability. The findings demonstrated that although TM had little impact on RWPE-1, it dramatically suppressed the growth of all PCa cells (Fig. 1A). TM exerted an apparent influence in androgen‐independent PCa cells (DU145 and PC-3) in both a dose‐dependent and time‐dependent manner (Fig. 1B, C). Therefore, we used these two cell lines to carry out a series of follow-up investigations. Additionally, we also observed TM obviously suppressed the colony formation ability of PCa cells (Fig. 1D, E).

Next, we explored whether TM could promote PCa cells apoptosis or result in cell cycle arrest as it slowed the development of PCa cells. Using DU145 and PC-3 cells labeled with PI and 7-AAD, we conducted a flow cytometry analysis. The results indicated that TM significantly increased the population of G0/G1 cells while reducing the number of cells in the S phase or G2/M phase, suggesting a block in the G1/S cell cycle phase (Fig. 1F, G). Besides, we found that, in comparison to the vehicle control group, the apoptotic rate of PCa cells treated with TM was considerably higher (Fig. 1H, I). These findings indicate that TM exerts powerful anti-tumor effects on PCa cells.

### TM repressed the migration and invasion of PCa cells in vitro

**Fig. 2:**
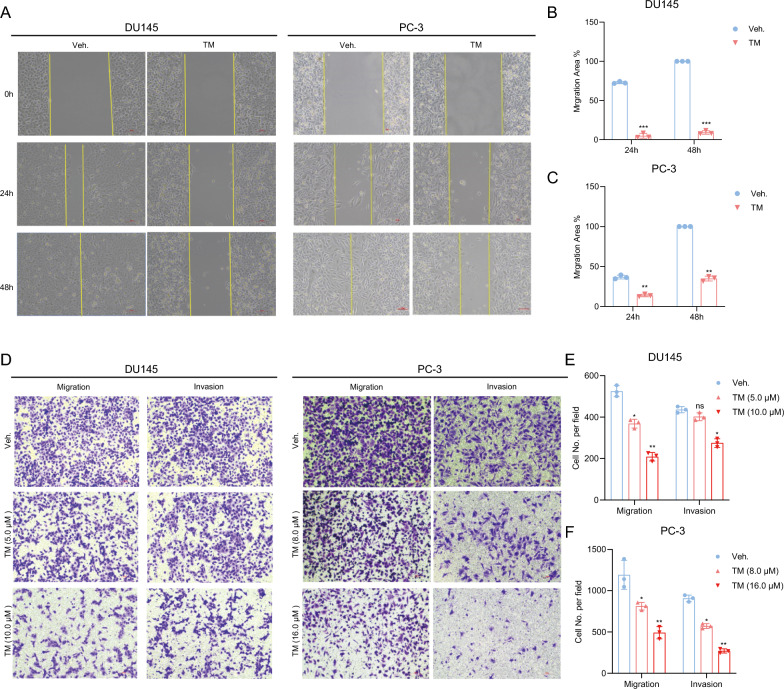
TM suppressed the capacity of migration and invasion in PCa cells. **A**–**C** Wound‐healing assays of DU145 and PC-3 cells treated with 5 μM and 8 μM TM, respectively, or vehicle. **D**–**F** Transwell migration and invasion experiments of DU145 and PC-3 cells treated with 5 and 10 μM, and 8 and 16 μM TM or vehicle respectively. Unpaired t test was used for the statistical analysis. *P < 0.05; **P < 0.01; ***P < 0.001; ns, no significance. Data are presented as mean ± SD of at least three independent experiments

To further test whether PCa cells’ ability of migration as well as invasion was suppressed by TM, we performed scratch experiments and transwell assays. It was observed that TM treatment significantly reduced the migratory ability of DU145 and PC-3 cells (Fig. 2A–C). Additionally, transwell migration tests revealed that TM inhibited cell mobility (Fig. 2D, E). We also performed transwell invasion assays to assess the impact of TM on the invasion of PCa cells and observed that the invasion capacity of PCa cells was significantly decreased in TM treatment groups compared to the vehicle control group as well (Fig. 2D, F). These findings suggest that TM effectively inhibits the migration and invasion of PCa cells in vitro.

### TM reduced the expression of GLI2 and inhibited SHH signaling

**Fig. 3:**
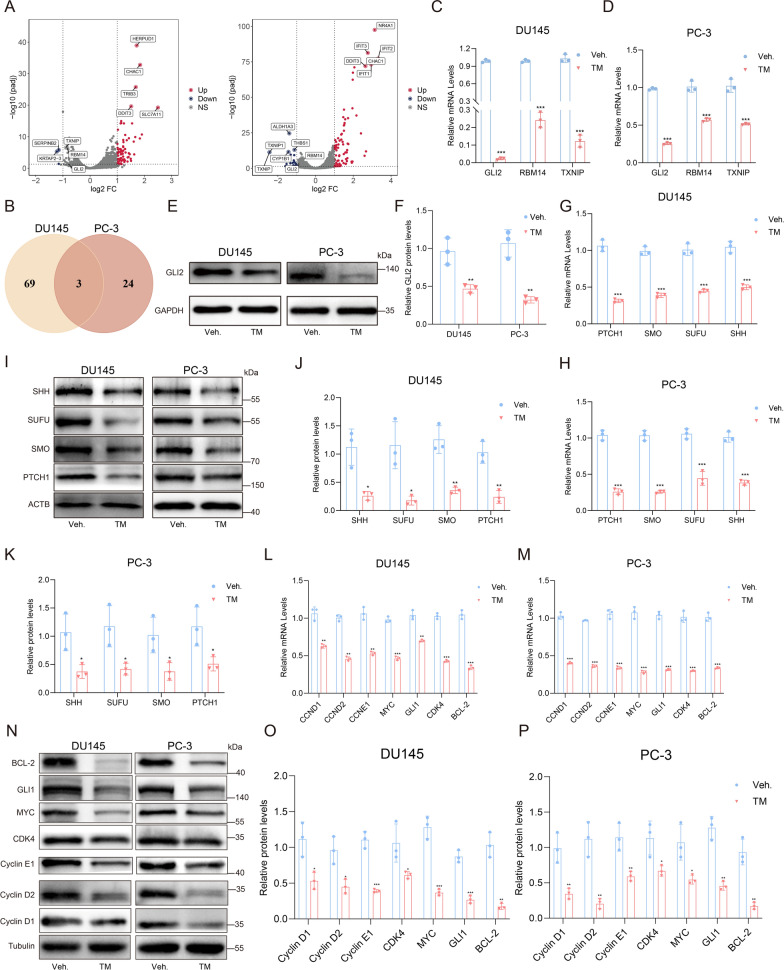
TM repressed activities of PCa cells via downregulating GLI2 and its target genes. **A** Scatterplot of RNA-seq data from DU145 and PC-3 cells treated with vehicle and TM. The upregulated (red) and downregulated (blue) genes at the translational level are highlighted. **B** The overlap of downregulated genes between DU145 and PC-3 cells. **C**, **D** RT-qPCR analysis of transcription levels of the common downregulated genes in DU145 and PC-3 cells. **E**, **F** Immunoblot analysis of GLI2 in DU145 and PC-3 cells treated with vehicle and TM. GAPDH was used as a loading control. **G**, **H** RT-qPCR analysis of transcription levels and **I**-**K** immunoblot analysis of SHH signaling pathway related proteins in DU145 and PC-3 cells treated with vehicle and TM. β-actin was used as a loading control. **L**, **M** RT**-**qPCR analysis of transcription levels of GLI2 target genes in DU145 and PC-3 cells treated with vehicle and TM. **N**–**P** Immunoblot analysis of GLI2 target genes in DU145 and PC-3 cells treated with vehicle and TM. β-tubulin was used as a loading control. Unpaired t test was used for the statistical analysis. *P < 0.05; **P < 0.01; ***P < 0.001; ns, no significance. Data are presented as mean ± SD of at least three independent experiments

In order to elucidate the mechanism behind TM's inhibitory effects on PCa cells, we used RNA-seq to assess the transcriptional factors that differed between DU145 and PC-3 cells treated with TM and vehicle. In DU145 and PC-3 cells, we detected 72 and 27 downregulated genes as well as 206 and 227 upregulated genes respectively (Fig. 3A). Then we focused on the downregulated genes between DU145 and PC-3 cells. After overlap, we obtained three genes, GLI2, RBM14, and TXNIP and performed RT-qPCR to verify their expression (Fig. 3B). The results were in line with the data from RNA-seq, and we found that GLI2 was the most significantly downregulated gene after TM treatment (Fig. 3C, D). Therefore, we listed GLI2 as the candidate gene and detected its protein level. It was found that the protein level of GLI2 in cells treated with TM was reduced significantly (Fig. 3E,F).

As previously reported, GLI2 is a transcription factor, which serves a crucial role in SHH signaling to mediate the development of cancers (Zhang et al. 2022; Chen et al. 2022; Gupta et al. 2019; Jeng et al. 2020). Based on this finding, we supposed TM inhibited the activities of PCa cells through the SHH pathway and detected the level of SHH-related proteins. Interestingly, we observed that the SHH signaling pathway-related proteins, including PTCH1, SMO, SUFU, and SHH, decreased apparently both at their mRNA and protein levels (Fig. 3G–K). As GLI2 functions by promoting the expression of its target genes consisting of Cyclin D, Cyclin E, Myc, and GLI1, we further detected these target genes and the results demonstrated that the expression of all these genes was reduced (Fig. 3L–P). Similarly, we also explored the expression of CDK4, which mediates the cell cycle, and Bcl-2, a protein that inhibits apoptosis. The results indicated that the levels of these two proteins were reduced as well. Taken together, TM could result in the downregulation of GLI2 along with its target genes and inhibit the SHH signaling pathway.

### GLI2 overexpression restored the effects caused by TM

**Fig. 4:**
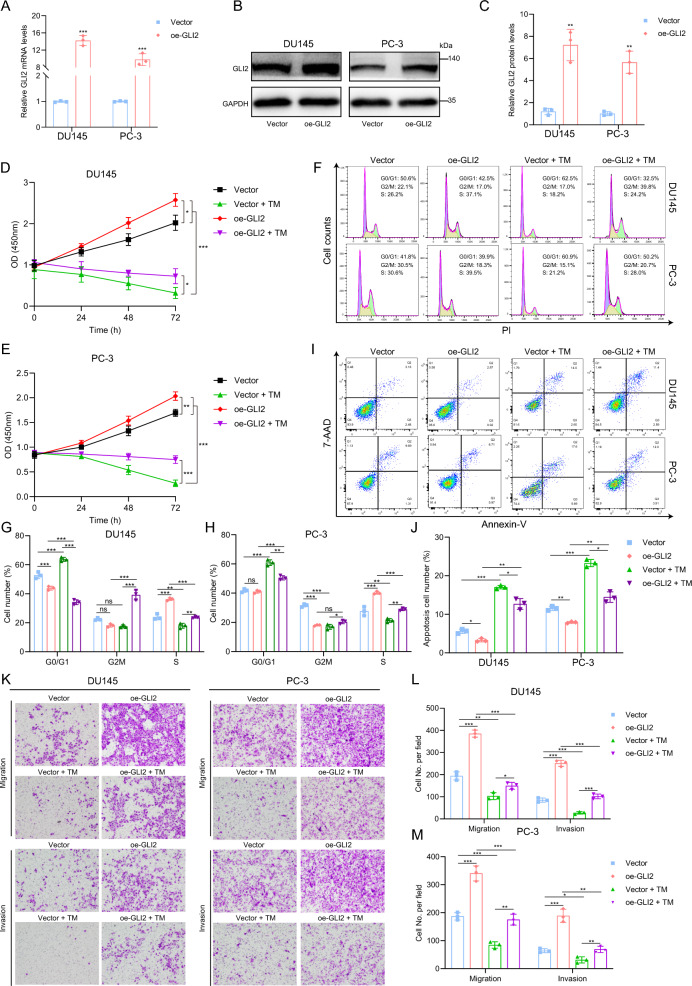
Overexpression of GLI2 restored the effects induced by TM. **A** RT-qPCR analysis of transcription levels of GLI2 in control and GLI2-overexpression DU145 and PC-3 cells. **B**, **C** Immunoblot analysis of GLI2 in control and GLI2-overexpression DU145 and PC-3 cells. **D**, **E** Cell proliferation analysis in control, GLI2-overexpression, control treated with TM and GLI2-overexpression treated with TM DU145 and PC-3 cells. **F**–**H** Cell cycle distribution in control, GLI2-overexpression, control treated with TM and GLI2-overexpression treated with TM DU145 and PC-3 cells. **I**, **J** Flow cytometry assays of the apoptotic percentage (including viable and non‐viable apoptotic cells) in in control, GLI2-overexpression, control treated with TM and GLI2-overexpression treated with TM DU145 and PC-3 cells. **K**–**M** Transwell migration and invasion experiments in control and GLI2-overexpression DU145 and PC-3 cells. Unpaired t test was used for the statistical analysis. *P < 0.05; **P < 0.01; ***P < 0.001; ns, no significance. Data are presented as mean ± SD of at least three independent experiments

To further determine the role of GLI2 in TM inhibition of PCa cells, we constructed DU145 (DU145^oe^) and PC-3 (PC-3^oe^) cells overexpressing GLI2 through lentivirus while cells infected with vector were selected as the control group (Fig. 4A–C). Subsequently, we treated DU145^oe^, PC-3^oe^ and control cells with TM and divided them into four groups, vehicle, vehicle treated with TM, GLI2-overexpression and GLI2 overexpression treated with TM, which were then detected activities through functional assays. The results demonstrated that GLI2 overexpression partially restored the inhibitory effect caused by TM (Fig. 4D–M). These findings indicate TM suppresses the activities of PCa cells and its target genes through downregulating GLI2.

### TM suppressed tumor growth in vivo

**Fig. 5:**
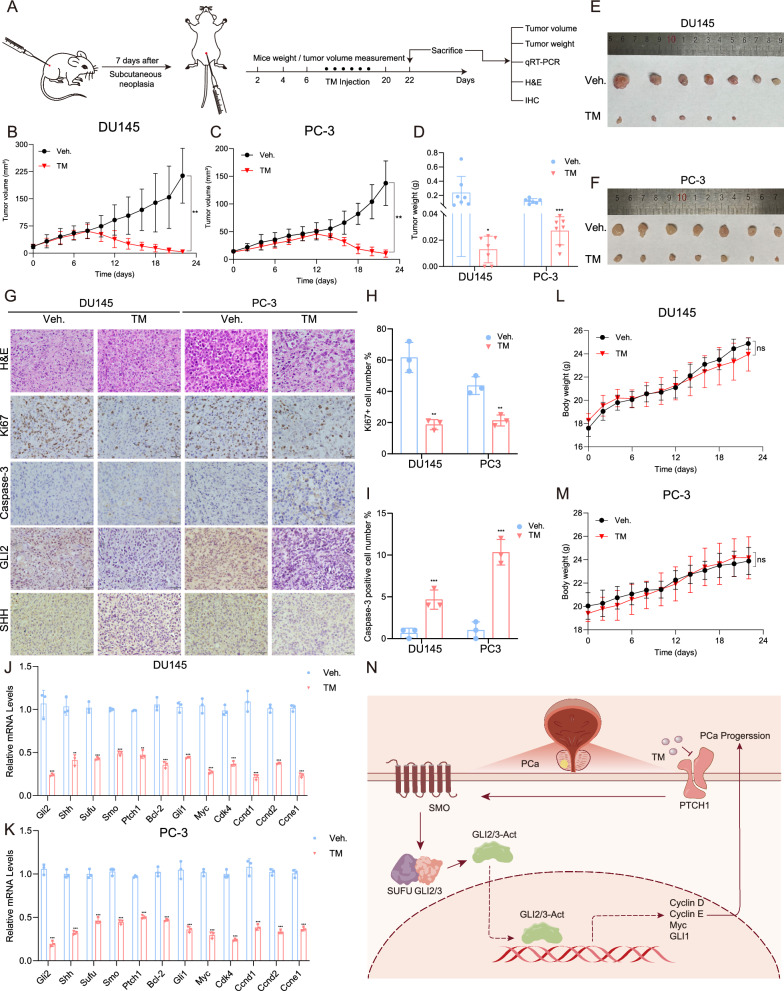
TM inhibited tumor growth in vivo. **A** Schematic of xenograft mouse model. Nude mice were subcutaneously injected with DU145 and PC-3 cells and intraperitoneally injected with TM. **B**, **C** Relative tumor volume in nude mice. Tumor volume was measured once every two days from the day of drug administration. **D** Weight of xenograft tumors when they were harvested. **E**, **F** Photographs of xenograft tumors when they were harvested. **G** H&E staining and IHC images with antibodies against Ki67, cleaved caspase‐3, GLI2 and SHH in DU145 and PC-3 xenograft tumors. **H** Proportion of Ki67 positive cells was reduced in xenograft tumors upon TM treatment. **I** Proportion of cleaved caspase‐3 positive cells was increased in xenograft tumors upon TM treatment. **J**, **K** RT-qPCR analysis of mRNA levels of key genes related to SHH signaling pathway in tumor tissue. **L**, **M** Body weight of nude mice. Body weight was measured once every two days from the day of drug administration. **N** A schematic model of the mechanism underlying the role of TM in PCa. Unpaired t test was used for the statistical analysis. *P < 0.05; **P < 0.01; ***P < 0.001. Data are presented as mean ± SD

To investigate whether TM exerted antitumor effects in vivo, we next used a nude mouse xenograft model (Fig. 5A). TM was discovered to greatly repress the development of xenograft tumors and some of the tumors were even not detectable (Fig. 5B–F). According to IHC results, TM treatment increased the percentage of cleaved caspase3 positive cells while decreasing the levels of GLI2, SHH, and Ki67, a proliferating cell marker (Fig. 5G–I). These results demonstrated that TM slowed the growth of PCa cells by causing apoptosis and blocking cell division in vivo. Next, we also detected the key genes related to the SHH signaling pathway and found that in tumors from mice treated with TM, these genes were downregulated at their mRNA levels significantly, which was consistent with the protein levels (Fig. 5J, K). Of note, there was no significance about body weight between mice treated with TM and vehicle, which indicated that TM showed little toxicity to mice (Fig. 5L, M). Taken together, these findings suggest that TM could suppress the growth of PCa cells in vivo.

## Discussion

In this work, we examined TM's possible anti-cancer effects, a drug once used for the treatment of IBS, but now used as an anti-cancer agent, on PCa cells. Our findings indicate that TM can significantly suppress proliferation, colony formation, migration, as well as invasion of PCa cells. Moreover, when administered systemically, TM considerably repressed the tumor growth of PCa cells in the mouse xenograft model. We also found that TM led to a reduction of GLI2 and inhibited the SHH signaling pathway, thus repressing the growth and promoting apoptosis of PCa cells.

Developing effective anti-cancer drugs is a challenging task (Kriplani and Guarve 2022; Ze et al. 2021). However, repositioning already existing clinical drugs could be an attractive solution, because of their in vivo safety, and pharmacokinetics and pharmacodynamics were well-understood (Tulsi et al. 2021). In this work, the effects of TM on multiple PCa cell lines, including PC-3, LNCaP, DU145, 22RV-1, and RWPE-1 normal prostate epithelial cells were investigated. Interestingly, we found that all PCa cells were sensitive to TM except RWPE-1. Moreover, TM treatment demonstrated great anti-tumor effects on androgen‐independent cells, DU145 and PC-3 cells. TM inhibited their proliferation, migration, and invasion, with very little damage to RWPE-1 cells in vitro. In the nude mouse xenograft model, TM showed great anti-tumor effects with little general toxicity. These findings suggest that TM could be an encouraging treatment for PCa. Additional studies are required to assess its possible therapeutic use and transfer it into clinical use.

RNA-seq analysis showed that after TM treatment, there was an obvious decrease in the transcription levels of GLI2, a transcriptional factor, that regulates the cancer cell proliferation and apoptosis in the SHH signaling pathway. The SHH signaling pathway is essential to the growth of malignancies. When the HH ligand combines with the receptor PTCH1, PTCH1 will be activated and signaling is transferred to downstream molecules including SMO, SUFU/GLI2, thus activating GLI2 and promoting the activated GLI2 into the nucleus. The increased activated GLI2 further accelerates the translation of its target genes. Excitingly, we found that TM could reduce the level of PTCH1 and its downstream proteins, therefore inhibiting the SHH signaling pathway (Fig. 5N). The RT-qPCR assays and Western blotting analysis further confirmed the decrease in the transcription and translation of GLI2, SHH-related proteins and GLI2’s target genes. These results suggest that TM may be a potential SHH-targeted PCa treatment drug. Through downregulating GLI2 as well as its target genes, and inhibiting the SHH signaling pathway, TM effectively inhibited the growth and promotes apoptosis of PCa cells.

TM is a drug that was initially approved by the FDA in 2002 for the treatment of constipated IBS, but was withdrawn in 2007 due to possible cardiovascular adverse effects (Sayuk and Tack 2022). In recent years, studies identified it as a novel anti-cancer drug for various cancers. A recent review of the clinical data has re-evaluated the cardiovascular safety profile of TM and drawn a conclusion that TM is safe for women < 65 years of age with IBS with constipation, no history of cardiovascular ischemic events, and ≤ 1 cardiovascular risk factor (Lacy et al. 2022). This indicates that TM may be re-introduced to treat IBS and even cancer. In our study, we examined its anti-cancer effects on PCa cells both in vitro and in vivo. Our study found that it showed inhibition in proliferation, migration and invasion, but prompted apoptosis in PCa cells in vitro. Additionally, we also found TM inhibited tumor growth in mouse models. We observed that there was no significance between mice treated with TM and vehicle, and speculated that TM exerts little toxicity to mice. However, whether it exerts other side effects such as cardiovascular adverse effects remains unknown, which needs to be evaluated and studied in future research.

As TM could effectively inhibit the SHH signaling pathway, TM could be a novel SHH inhibitor and be used to treat diseases caused by the activation of the SHH signaling pathway. Future attention could be paid to investigations about this and transfer it into clinical applications.

## Conclusion

In summary, our study demonstrated that TM could inhibit the progression of PCa cells. The drug not only suppressed proliferation but also reduced migration as well as invasion in PCa cells. Additionally, TM inhibited the development of tumors in vivo. These findings demonstrate TM's potential as an anti-cancer medication for PCa therapy. TM may be a promising antagonist of the SHH signaling pathway, thus being used to treat SHH-related diseases.

## Supplementary Information

Supplementary Material 1.

## Data Availability

The data that support the findings of this study are available from the corresponding author upon reasonable request.
