# Supplementary material for: Tegaserod maleate exerts anti-tumor effects on prostate cancer via repressing sonic hedgehog signaling pathway
Source: Mol Med. 2025 Jan 29;31:30. doi: 10.1186/s10020-025-01080-1 (PMC11780919; doi:10.1186/s10020-025-01080-1)
Supplement: Supplementary file 1 — Supplementary Material 1. [file 10020_2025_1080_MOESM1_ESM.docx]

**Appendix A. Supplementary data**

**Table 1. Primer sequence of RT-qPCR.**

| **Gene** | **Forward sequence** | **Reverse sequence** |
| --- | --- | --- |
| h-GLI2 | CTGCCTCCGAGAAGCAAGAAG | GCATGGAATGGTGGCAAGAG |
| h-RBM14 | CCCCGAGCCTCTTATGTGG | GTCATGGGCTGAGTCCGATAG |
| h-TXNIP | ATATGGGTGTGTAGACTACTGGG | GCAGGTACTCCGAAGTCTGT |
| h-PTCH1 | ACTTCAAGGGGTACGAGTATGT | TGCGACACTCTGATGAACCAC |
| h-SMO | TCGAATCGCTACCCTGCTG | CAAGCCTCATGGTGCCATCT |
| h-SUFU | CACGCCATCTACGGAGAGTG | GTACTTGACGATAGCGGTAACC |
| h-SHH | CTCGCTGCTGGTATGCTCG | ATCGCTCGGAGTTTCTGGAGA |
| h-CCND1 | GCTGCGAAGTGGAAACCATC | CCTCCTTCTGCACACATTTGAA |
| h-CCND2 | CTGTCTCTGATCCGCAAGCAT | GGTGGGTACATGGCAAACTTAAA |
| h-CCNE1 | AAGGAGCGGGACACCATGA | ACGGTCACGTTTGCCTTCC |
| h-MYC | GGCTCCTGGCAAAAGGTCA | CTGCGTAGTTGTGCTGATGT |
| h-GLI1 | AGCGTGAGCCTGAATCTGTG | CAGCATGTACTGGGCTTTGAA |
| h-CDK4 | TCAGCACAGTTCGTGAGGTG | GTCCATCAGCCGGACAACAT |
| h-BCL-2 | GGTGGGGTCATGTGTGTGG | CGGTTCAGGTACTCAGTCATCC |
| h-GAPDH | ATCACTGCCACCCAGAAGAC | TTTCTAGACGGCAGGTCAGG |
| m-Gli2 | GCCCTGGAGAGTCACCCTT | TGCACAGACCGGAGGTAGT |
| m-Shh | AAAGCTGACCCCTTTAGCCTA | TTCGGAGTTTCTTGTGATCTTCC |
| m-Sufu | GGGACTGCACGCCATCTAC | TTGACGATAGCGGTAACCTGG |
| m-Smo | GAGCGTAGCTTCCGGGACTA | CTGGGCCGATTCTTGATCTCA |
| m-Ptch1 | GCCTTCGCTGTGGGATTAAAG | CTTCTCCTATCTTCTGACGGGT |
| m-Bcl-2 | CCTGTGGATGACTGAGTACCTG | AGCCAGGAGAAATCAAACAGAGG |
| m-Gli1 | AACGCTATACAGATCCTAGCTCG | GTGCCGTTTGGTCACATGG |
| m-Myc | ATGCCCCTCAACGTGAACTTC | CGCAACATAGGATGGAGAGCA |
| m-Cdk4 | ATGGCTGCCACTCGATATGAA | TCCTCCATTAGGAACTCTCACAC |
| m-Ccnd1 | GCGTACCCTGACACCAATCTC | CTCCTCTTCGCACTTCTGCTC |
| m-Ccnd2 | GAGTGGGAACTGGTAGTGTTG | CGCACAGAGCGATGAAGGT |
| m-Ccne1 | GTGGCTCCGACCTTTCAGTC | CACAGTCTTGTCAATCTTGGCA |
| m-Gapdh | AGGTCGGTGTGAACGGATTTG | GGGGTCGTTGATGGCAACA |
